# Repair of partial atrioventricular canal defect in adult patients: two-year follow-up outcomes of a retrospective study

**DOI:** 10.1186/s13019-019-0931-x

**Published:** 2019-06-11

**Authors:** Lingyun Song, Yunfei Ling, Qi An

**Affiliations:** Department of Cardiovascular Surgery, West China Hospital, Sichuan University, No. 37 GuoXue Xiang, Chengdu, Sichuan 610041 People’s Republic of China

**Keywords:** Partial atrioventricular canal defect, Congenital heart disease, Partial atrioventricular septal defect, Cardiovascular surgery, Retrospective study

## Abstract

**Background:**

Partial atrioventricular canal defects (PAVC) are preferred to be repaired when diagnosed and before an operation would interfere with school. There were rare previous studies about partial atrioventricular canal defect operations in adult patients. In this single-center retrospective study, we mean to review the mid-term follow-up outcomes of late diagnosed and repaired partial atrioventricular canal defects in adult patients.

**Methods:**

46 adult partial atrioventricular canal defect patients who underwent operation in West China Medical Center from 2009 to 2017 were included. Required data were obtained from operation notes, patient charts and the outpatient records.

**Results:**

Among 46 patients, 10(21.7%)were male and mean age at operation was 37.6 ± 12.4 years. 11 patients had prior arrythmia, including 8 atrial fibrillations, 2 atrioventricular blocks and 1 left bundle branch block. There were 41 patients with tricuspid valve regurgitation and 22 underwent tricuspid valvuloplasty. All the patients had mitral regurgitation. 6 patients with valve incrassation and shrinkage underwent mitral valve replacement, and the rest underwent mitral repair surgery. There was one early death post operation and no more mortalities in the following follow-up years. According to the follow-up outcomes, heart function of the patients recovered significantly, dilation of atriums and ventricles, except for left atriums, were reversed to a large extent and all but one patients’ tricuspid valve regurgitations were reduced to mild and below. 4(8.7%) patients underwent reoperation and the main reasons were arrythmia and recurrent severe mitral valve regurgitation.

**Conclusion:**

Partial atrioventricular canal defect repair in adult patients can achieve good results. Compared with the results of patients underwent operations in preschool years, though delayed surgery timing seems to bring more preoperative complications and influences heart function, the mortality and reoperation rate are excellent.

## Introduction

The spectrum of atrioventricular septal defects account for about 7–17% of congenital heart disease [1], and 25% of them are partial atrioventricular canal defects [2]. The repairs of PAVC are preferred to be performed when diagnosed and before an operation might interfere with school [3]. According to the long-term follow-up results of other centers, surgical outcomes were excellent [3, 4]. There were many reports about the surgery outcomes of patients in young age. However, there is a lack of report involving surgery outcomes in adult patients. The aim of this retrospective study is to review the results of treating adult PAVC patients in our center. The mortality rate, reoperation rate, surgery procedures and valve regurgitation associated data were described.

## Methods

The retrospective study was designed to collect data of the adult patients diagnosed with PAVC and received surgery repair in West China Hospital from 01.01.2009–01.11.2011. Among 52 patients, 6 who failed to continue follow-up because of their living so far away and economic reasons were excluded, and there were finally 46 patients enrolled in the study. In this retrospective study, operation notes, patient charts, intensive care unit patient records, echocardiography outcomes, outpatient records and all the applicable data were searched for required information. A database was created to record all the useful information of each patient as the following dataset: hospital number, gender, weight at surgery, age at surgery, follow-up years, diagnosis, surgical procedure, preoperative degree of valve regurgitation, preoperative arrythmia, echocardiographic size of atrium and ventricle, prior surgery, presence of concomitant diagnosis, cardiopulmonary bypass times, aortic cross-clamp times, length of hospital stay, residual complications post operation, reoperation, most recent echocardiographic findings and survival status. The content of surgical procedure included the type of mitral valve repair, the repair of atrial septal defect, the type of tricuspid valvuloplasty and maze procedure in patients with preoperative atrial fibrillation. The severity of valve regurgitation was described by the ultrasonologists who distinguished the regurgitations into none/trace, mild, moderate and severe four levels according to the color flow doppler appearance of the components of the valve regurgitation jet from multiple views. The diameter of atriums and ventricles were also evaluated via transthoracic echocardiography. The patients were evaluated by the same echo team prior to surgery, postoperatively and during the follow-up. There were 4 echo clinicians in the team and for each patient’s evaluation, at least two of them made a decision together.

### Statistical analysis

Data were analyzed with IBM SPSS Statistics Version 21.0 (SPSS Inc., Chicago, IL, USA). Distribution of the continuous variables were assessed, Shapiro-Wilks test were used. Continuous variables were presented as mean+/− standard deviation or median and interquartile range according to the distribution of the variables. Categorical data were presented as counts and frequencies. Standard descriptive statistics were used to summarize the data. To compare differences between groups, we used t-test for continuous variables, and Pearson’s chi-squared test for categorical variables. And the threshold for statistical significance was taken as *P <* 0.05.

## Results

### Preoperative characteristics

During the study period, there were finally 46 patients enrolled in the study, of which 10(21.7%) were male patients. The characteristics of all the patients are summarized in Table 1. The mean weight and mean age at surgery were 53.1 ± 8.7 kg and 37.6 ± 12.4 years respectively. Besides ASD and mitral valve regurgitation, most adult PAVC patients had developed tricuspid regurgitation and some had developed arrythmia. There were 41 patients with TR, among which 21(45.7%) were mild, 15(32.6%) were moderate and 5(10.9%) were severe. All patients had MR, and 15(32.6%) were mild, 19(41.3%) were moderate and 12(26.1%) were severe. 8(17.4%) patients had atrial fibrillation, 2(4.3%) had third degree A-V block and 1(2.2%) had left bundle branch block. Transthoracic echocardiography demonstrated that the mean left atrial diastolic diameter was 40.1 ± 11.1 mm, the mean right atrial diastolic diameter was 54.2 ± 12.1 mm and the mean right ventricular diastolic diameter was 35.4 ± 6.4 mm. They all significantly exceeded reference value according to the result of t-test (*P* < 0.05) [5, 6]. However, it witnessed no dilatation of left ventricle with mean diastolic diameter of 42.3 ± 7.1 mm. According to NYHA (New York Heart Association) classification, the patients’ functional capabilities were divided into four classes, that was 5(10.9%) class I patients, 24(52.2%) class II patients, 16(34.8%) class III patients and 1(2.2%) class IV patient.

**Table 1:** Preoperative data

Variables	N(%)
Male	10(21.7%)
Weight at operation(mean ± SD)	53.1 ± 8.7 kg
Age at operation(mean ± SD)	37.6 ± 12.4 years
MR degree before operation	
Mild	15(32.6%)
Moderate	19(41.3%)
Severe	12(26.1%)
TR degree before operation	
Trace/none	5(10.9%)
Mild	21(45.7%)
Moderate	15(32.6%)
Severe	5(10.9%)
Arrythmia	
Atrial fibrillation	8(17.4%)
A-V block III	2(4.3%)
Left bundle branch block	1(2.2%)
NYHA class I	5(10.9%)
II	24(52.2%)
III	16(34.8%)
IV	1(2.2%)

*SD* standard deviation, *MR* mitral valve regurgitation, *TR* tricuspid valve regurgitation, *A-V* atrioventricular, *NYHA* New York Heart Association

### Operative procedures and details

The operations of the 46 patients were mainly performed by 2 experienced surgeons (5 operations were by another two surgeons). Almost all operations were performed via median sternotomy except for two thoracoscopic PAVC repair surgeries. Cardiopulmonary bypass was routinely instituted via direct bicaval and aortic cannulation for each patient and St. Thomas cardioplegia was used at 20-min intervals as cold antegrade perfusion to protect myocardium during the surgery. Mean CBP time and mean cross-clap time were 73.3 ± 13.0 min and 48.8 ± 12.8 min respectively**(**Table 2**)**. Mean minimal venous blood temperature during the cross-clap period was 32 ± 3.1 °C. Except for one suture closure, all the primum atrial septal defects were repaired with patch, 24(52.2%) patients used 0.6% glutaraldehyde soaked autologous pericardium patch, 10(21.7%) used Darcon patch and 11(23.9%) used tissue engineered bovine pericardium patch of Bal Medic. 10 patients had secundum ASDs, of which 1 was repaired with 0.6% glutaraldehyde soaked autologous pericardium, 2 were repaired with bovine pericardium and the rest were repaired with suture closure. There were 9 patients with PFOs and they were repaired with suture closure. All patch repaired ASDs were closed with McGoon procedure to protect the conducting bundles and there were no A-V block caused by the surgery. Most MRs were caused by leaflet cleft and some were caused by leaflet cleft and annular dilation. 40(87%) patients’ mitral valve clefts were repaired with intermittment suture, of which 6(13%) underwent mitral valve ring annuloplasty and 1 underwent posterior leaflet extension simultaneously. 6(13%) patients received mitral valve replacement though we tried valve repairs which ended in >moderate regurgitations because of valve incrassation and shrinkage. All mitral valve replacements were done prior to chest closure. 9(19.6%) patients underwent De Vega valvuloplasty for tricuspid valve regurgitation while 13 (28.3%) patients received ring annuloplasty. 2(4.3%) patients had muscular VSDs and both were repaired with suture closure. One patient underwent CoA(Coarctation of aorta) balloon dilatation prior to the PAVC repair.

**Table 2:** Operative details

Variables	N(%)
Operative procedure	
PAVC repair(primum ASD repair+ MV cleft suture)	40(87%)
+ secundum ASD repair	10(21.7%)
+ muscular VSD repair	2(4.3%)
+ TVP	17(40.0%)
+ MV ring plasty	6(13%)
+ PFO closure	9(19.6)
+ MAZE	3(6.5%)
+ Left atrium reduction	1(2.2%)
MVR+ primum ASD repair	6(13%)
+ MAZE	1(2.2%)
+ TVP	5(10.9%)
Primum ASD patch type	
Autologous pericardium patch	24(52.2)%
Darcon patch	10(21.7%)
Bovine pericardium patch	11(23.9%)
Cross-clamp time	48.8 ± 12.8 min
CPB time	73.3 ± 13.0 min

*PAVC* partial atrioventricular canal defect, *ASD* atrial septal defect, *VSD* ventricular septal defect, *TVP* tricuspid valvuloplasty, *MV* mitral valve, *MAZE* maze radiofrequency, *MVR* mitral valve replacement, *PFO* primary oval foramen, *CPB* cardiopulmonary bypass;”+” means simultaneously

### Post-operative details

The average collective duration of hospital stay was 15.0 ± 5.6 days. During this period, two patients still had atrial fibrillation which existed prior to surgery. Both patients received a cardioversion in operation but not successful. One patient with peri-operative A-V block and one with peri-operative left bundle branch block required pacemaker placement. Patients received proper treatment for the arrythmia. A patient had recurrent severe mitral valve regurgitation 2 days after the primary operation and received re-operation. We found three intermittent stiches on edge of the cleft teared off and repaired the cleft again and finally this patient discharged with mild regurgitation. One patient died for malignant ventricular arrythmia about 10 h after the surgery. The patient had atrial fibrillation prior to surgery and received successful mitral valve replacement, tricuspid repair and cardioversion. After the surgery, the patient had mild MR and mild TR and no longer had atrial fibrillation. The reason for the malignant arrythmia was not clear **(**Table 3**)**. Every patient was routinely supported with mechanical ventilation, vasoactive drugs, diuretics and antibiotics if necessary. There were no severe infections, some patients had mild or moderate infections which recovered quickly after appropriate treatment and did not interfere with discharge.

**Table 3:** Post-operative and follow-up details

Variables	N(%)
Length of hospital stay	15.0 ± 5.6 days
Residual complications	
Atrial fibrillation	2(4.4%)
Left bundle branch block	1(2.2%)
Third degree atrioventricular block	1(2.2%)
Most recent follow-up echo MR	
Trace/none	15(33.3%)
Mild	25(55.6%)
Moderate	3(6.7%)
severe	2(4.4%)
Most recent follow-up echo TR	
Trace/none	33(73.3%)
Mild	11(24.4%)
Moderate	1(2.2%)
Most recent follow-up heart function	
NYHA class I	34(75.6%)
NYHA class II	11(24.4%)
Mean follow-up years	2.96 ± 2.08 years
Early mortality	1(2.2%)
Late mortality	0(0%)
Reoperation	4(8.9%)
Permanent pacemaker implantation	2(4.4%)
Mitral valve replacement	1(2.2%)
Mitral repair	1(2.2%)

*MR* mitral valve regurgitation, *TR* tricuspid valve regurgitation, *NYHA* New York Heart Association

### Follow-up and reintervention

**Fig. 1:**
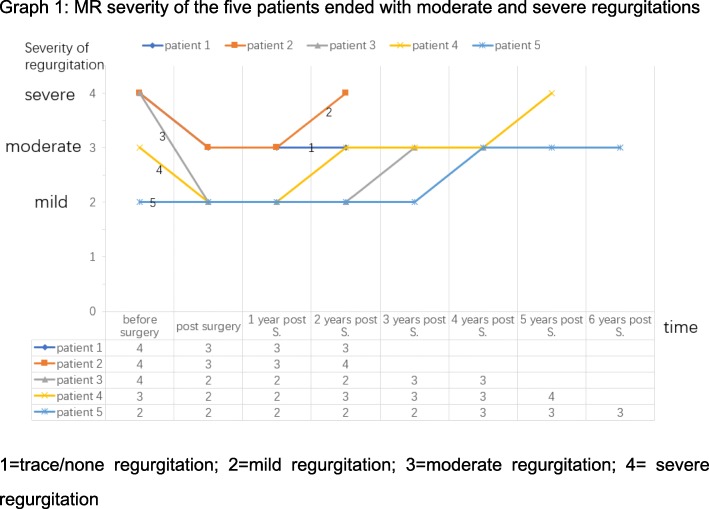
MR severity of the five patients ended with moderate and severe regurgitations. 1 = trace/none regurgitation; 2 = mild regurgitation; 3 = moderate regurgitation; 4 = severe regurgitation

**Fig. 2:**
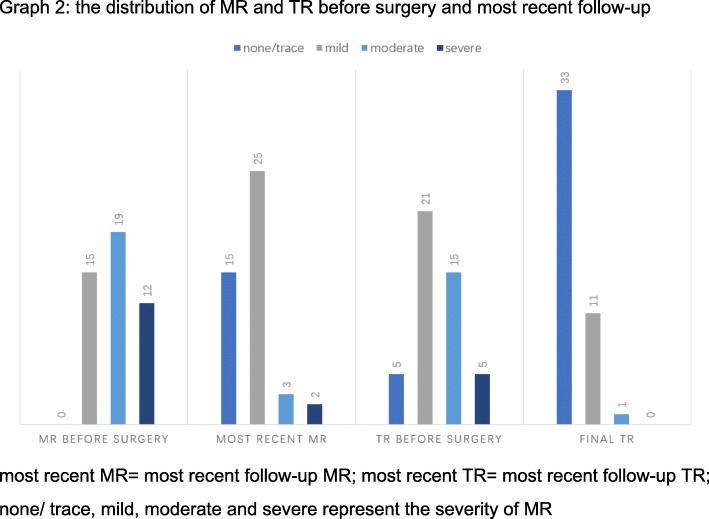
the distribution of MR and TR before surgery and most recent follow-up most recent MR = most recent follow-up MR; most recent TR = most recent follow-up TR; none/ trace, mild, moderate and severe represent the severity of MR.

**Fig. 3:**
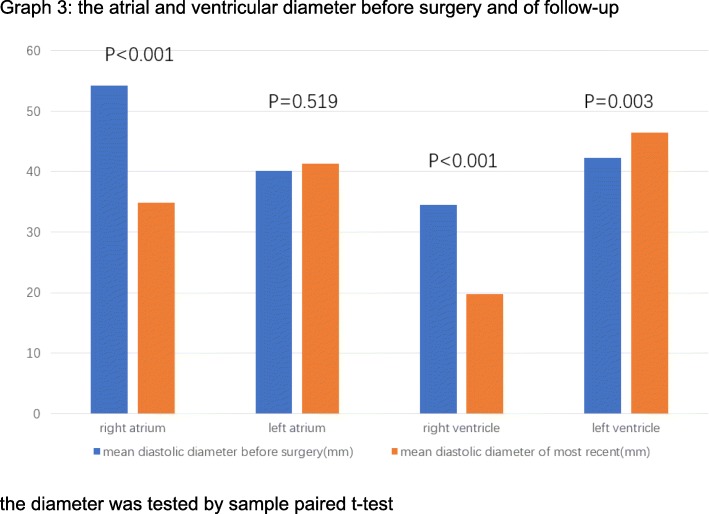
the atrial and ventricular diameter before surgery and of follow-up. the diameter was tested by sample paired t-test

The mean follow-up year was 2.96 ± 2.08 years. There was one early mortality and no late mortalities. One patient gained pericardial effusion 1 month after the surgery and underwent pericardiocentesis and drainage tube placement. Most recent echocardiographic findings about mitral valve revealed that there were 25(55.6%) patients with mild regurgitation, 3(6.7%) patients with moderate regurgitation and 2(4.4%) with severe regurgitation (Fig. **1**). As for tricuspid valve regurgitation according to follow-up data, there were 33(73.3%) patients with none or trace regurgitations, 11(24.4%) with mild regurgitations and 1(2.2%) with moderate regurgitations (Fig. 2). The mitral regurgitations as well as the tricuspid regurgitations were significantly improved compared to preoperative situation (*P* < 0.05, tested by *Pearson*’s chi-squared test). Compared with preoperative NYHA functional classes, there were 34(75.6%) patients in class Iand 11(24.4%) patients in class II, which improved significantly(*P* < 0.05, tested by *Pearson*’s chi-squared test). It was found during follow-up that mean diameter of diastolic left ventricles relatively dilated to 46.5 ± 5.0 mm, which was 4.10 ± 8.14 mm larger than the preoperative size (*P* = 0.003)and still with normal limits. Mean diameter of diastolic right atriums and diastolic right ventricles recovered to 37.8 ± 7.5 mm and 19.8 ± 8.2 mm, decreased by 16.83 ± 11.83 mm (*P* < 0.001) and 15.62 ± 8.17 mm (*P* < 0.001) respectively**(**Table 4**)**. But the mean diameter of diastolic left atriums was 41.3 ± 8.5 mm with no significant reduction(*P* = 0.519) (Fig. **3**). A patient who received primary mitral repair underwent secondary mitral valve replacement for recurrent severe mitral valve regurgitation after discharge. Two patients underwent permanent pacemaker implantation for third degree A-V block and left bundle branch block. Both were transvenous. And all the reoperation patients had good follow-up outcomes with <moderate MRs and NYHA class I functional capabilities till the end of the retrospective study.

**Table 4:** pre- and post-operation heart measurements

	Pre-operation measurements	Post-operation follow-up measurements	*P* value
Right atrium (mean ± SD)	54.2 ± 12.1 mm	37.8 ± 7.5 mm	< 0.001
Right ventricle (mean ± SD)	34.5 ± 6.4 mm	19.8 ± 8.2 mm	< 0.001
Left atrium (mean ± SD)	40.1 ± 11.1 mm	41.3 ± 8.5 mm	0.519
Left ventricle (mean ± SD)	42.3 ± 7.1 mm	46.5 ± 5.0 mm	0.003

*SD* standard deviation, paired samples t-test were used to calculate *P* value

## Discussion

Atrioventricular septal defects (AVSDs) are common among congenital heart diseases and they account for 3% of all major congenital cardiac defects. AVSDs are classified into three categories: complete, partial and transitional [7]. Operation via median sternotomy to repair AVSDs was first introduced in 1955 [8]. As more than 60 years passed, the surgical technique and medical technology have advanced [9]. Partial atrioventricular canal defect repair is relatively mature now. In open heart surgery, the surgical procedures mainly encompass mitral valve cleft suture and primum atrial septal defect repair [3, 10, 11]. According to the reports, most patients only need the two procedures in surgery and the survival following surgeries revealed excellent results.

The optimal timing of elective surgery repair remains controversial. Although different centers have not come to an agreement, most of the reports state that the optimal time for repair is during the early childhood [4, 10]. Therefore, most patients undergo operation in childhood. There is a lack of reports about the outcomes of patients who undergo PAVC operation when they are already adults. This retrospective study was aimed at reviewing the mid-term survival, reoperation incidence and complications caused by delayed operation in adult PAVC patients.

A long-term retrospective study by Najdawi et al. reported a 2% mortality rate in 30 days and a 6% mortality rate 5 years after surgery [4]. A single-institute retrospective study including 86 patients demonstrated two deaths in long-term follow-up [11]. Most of the studies about PAVC operation revealed good follow-up results [3, 4, 12, 13]. In our study, there was no late mortality. One patient died at the night of operation because of malignant ventricular arrythmia. The mortality rate was 2.2%. Though delayed diagnoses and treatments, results of our study were very good.

During the follow-up period, 4(8.7%) patients need reinterventions: 2 for permanent pacemaker implantation and 2 for recurrent severe mitral valve regurgitation. In published studies, the reoperation rates ranged around 10–15% [3, 4, 10, 11, 13]. The reoperation rate in this study was acceptable. The reasons for reoperations or reinterventions in adult patients were different from those of young children. According to the reports, the reasons leading to reoperations in patients who received surgeries in childhood were most likely to be left ventricular outflow tract obstructions, mitral valve insufficiency or stenosis, residual atrial septal defects or the implantations of pacemakers [4, 11, 14]. For patients who underwent operations in adulthood, the main reasons were recurrent severe mitral valve regurgitations and implantations of permanent pacemakers [15, 16].

The mortality and reoperation rate were excellent. However, the delayed operations gave rise to additional complications. There were few PAVCs combined with tricuspid valve regurgitations in previous reports about patients underwent operations in childhood [10, 11]. In our study, there were 41(89.1%) patients had tricuspid valve regurgitations, of which 20(43.5%) were moderate or severe. 22(47.8%) patients underwent tricuspid repair in PAVC repair surgery simultaneously. The follow-up outcomes showed there were only one patient still having moderate regurgitation and none had severe regurgitation any longer. Tricuspid valvuloplasty is very effective in adult PAVSD patients.

According to the result, the mean diastolic diameter of left atriums, right atriums and right ventricles obviously increased, while the mean diastolic diameter of left ventricles stayed normal. Patients’ preoperative NYHA classes decreased significantly to class II(24, 52.6%), class III(16, 34.8%) and class IV(1, 2.2%)(*P* < 0.001, tested by *Pearson*’s chi-squared test). Arrythmia especially atrial fibrillations were common among these patients. On the other hand, there were few reports demonstrating significant atriums’ or ventricles’ dilation in young partial AVSD patients, and the arrythmias were relatively infrequent [10, 11]. Delayed operations seemed to induce additional preoperative complications in patients. Post operation, according to most recent result, the condition of tricuspid valve regurgitations, dilated right atriums and right ventricles and arrythmia were all improved. However, dilation of left atriums saw no change, which had influence on heart function [17]. The reason and mechanism behind and the long-term effect of dilated left atriums on postoperative adult PAVC patients need further studies.

## Conclusion

Partial atrioventricular canal defect repair operation in adult patients can achieve good results. Compared with the results of patients underwent operations in preschool years, though delayed surgery timing seems to bring more preoperative complications and influences heart function, the mortality and reoperation rate are excellent.

## Data Availability

All data generated or analyzed during this study are included in this published article [and its supplementary information files].

## Abbreviations

ASD: Atrial septal defect
A-V block: Atrioventricular block
CoA: Coarctation of aorta
MR: Mitral valve regurgitation
PAVC: Partial atrioventricular canal defect
TR: Tricuspid valve regurgitation
VSD: Ventricular septal defect
